# Educational attainment and mental health outcomes: A within-sibship Mendelian randomization study

**DOI:** 10.1017/S0033291725101736

**Published:** 2025-09-26

**Authors:** María Fernanda Vinueza Veloz, Laxmi Bhatta, Paul R. Jones, Martin Tesli, George Davey Smith, Neil M. Davies, Ben M. Brumpton, Øyvind E. Næss

**Affiliations:** 1 Department of Community Medicine and Global Health, Institute of Health and Society, Faculty of Medicine, University of Oslo, Oslo, Norway; 2 K.G. Jebsen Center for Genetic Epidemiology, Department of Public Health and Nursing, Norwegian University of Science and Technology – NTNU, Trondheim, Norway; 3 FIU-PH, Division of Mental Health Care, St Olavs Hospital, Trondheim, Norway; 4 Department of Mental Disorders, Norwegian Institute of Public Health, Oslo, Norway; 5 Centre for Research and Education in Forensic Psychiatry, Department of Mental Health and Addiction, Oslo University Hospital, Oslo, Norway; 6 Department of Adult Psychiatry, Diakonhjemmet Hospital, Oslo, Norway; 7 Department of Psychiatry, Østfold Hospital, Grålum, Norway; 8 MRC Integrative Epidemiology Unit (IEU), University of Bristol, Bristol, UK; 9 Division of Psychiatry, University College London, London, UK; 10 Department of Statistical Sciences, University College London, London, UK; 11 HUNT Research Center, Department of Public and Nursing, Norwegian University of Science and Technology – NTNU, Trondheim, Norway; 12 Clinic of Medicine, St. Olavs Hospital, Trondheim University Hospital, Trondheim, Norway; 13 Department Chronic diseases, Norwegian Institute of Public Health, Oslo, Norway

**Keywords:** anxiety, depression, educational attainment, Mendelian randomization, neuroticism, psychotropic medication

## Abstract

**Background:**

Observational studies indicate that higher educational attainment (EA) is associated with a lower risk of many mental health conditions (MHC). We assessed to what extent this association is influenced by genetic nurture and demographic factors (i.e., assortative mating and population structure).

**Methods:**

We conducted a within-sibship Mendelian randomization (MR) study. The sample consisted of 61 880 siblings (27 507 sibships) from the Trøndelag Health Study-HUNT (Norway) and UK Biobank (United Kingdom). MHC outcomes included symptom scores for anxiety, depression, and neuroticism, measured using the Hospital Anxiety and Depression Scale, the 7-item Generalized Anxiety Disorder Scale, the 9-item Patient Health Questionnaire, and the Eysenck Personality Questionnaire, along with self-reported psychotropic medication use.

**Results:**

One standard deviation (SD) increase in liability to EA was associated with lower anxiety (−0.20 SD [95% CI: −0.26, −0.14]), depression (−0.11 SD [−0.43, −0.22]), and neuroticism scores (−0.30 SD [−0.53, −0.06]), as well as lower odds of psychotropic medication use (OR: 0.60 [0.52, 0.69]). Within-sibship MR estimates remained consistent with population-based estimates: anxiety (−0.17 SD [−0.33, −0.00]); depression (−0.18 SD [−1.26, 0.89]); neuroticism (−0.29 SD [−0.43, −0.15]); psychotropic medication use (OR, 0.52 [0.34, 0.82]).

**Conclusions:**

Higher EA or genetic liability to education reduces symptoms of anxiety, neuroticism, and psychotropic medication use. These mental health benefits do not seem to be explained by EA-linked genetic nurture or demographic factors. Regarding depression, results were less conclusive due to imprecise estimates, though beneficial effects of genetic liability to higher EA are possible and warrant further investigation.

## Introduction

Mental health conditions (MHC), which are defined as mental disorders, psychosocial disabilities, and mental states associated with significant distress, impaired functioning, or risk of self-harm, represent a major global health burden (World Health Organization, 2022). Common MHC such as anxiety and mood disorders (e.g., depression) are among the leading causes of years lived with disability across all age groups (Abbafati et al., 2020). Furthermore, individuals with MHC face mortality rates at least twice as high as those of the general population or individuals without these conditions (Walker et al., 2015).

Socioeconomic factors, including those related to education, are strongly implicated in the risk of developing MHC (Kivimäki et al., 2020). Higher educational attainment (EA) is associated with a reduced risk of developing MHC such as anxiety, depression, personality disorders, and substance abuse (Bjelland et al., 2008; Cohen et al., 2020; Erickson et al., 2016). This inverse relationship may be attributed to greater access to mental and physical resources in adulthood, which help educated individuals cope with adversity and mitigate the development of MHC (Niemeyer et al., 2019).

Mendelian randomization (MR) is a method that uses genetic variants or polygenic scores (PGS) as instrumental variables to investigate relationships between exposures and outcomes. MR relies on the premise that there is a causal pathway from an individual’s genotype to the individual’s phenotype (e.g., a genotype–phenotype association). MR is robust to exposure-outcome confounding and reverse causation because it leverages the principle that genetic variants are randomly assigned at conception, and do not change throughout life (Davies et al., 2018; Evans & Davey Smith, 2015; Smith & Ebrahim, 2003).

Valid causal inference using MR requires that three core instrumental variable assumptions hold: (i) The genetic variants must be associated to the exposure of interest and this relationship is required to be reasonably strong (relevance). (ii) There must be no uncontrolled common causes of the genetic variants and the outcome (independence). (iii) The genetic variants must influence the outcome solely through the exposure of interest, which implies that the effect of the genetic variants on the outcome is fully mediated by the exposure (exclusion) (Walker et al., 2024).

Two recent population-based MR studies support the role of EA in the etiology of common MHC, including anxiety and depression (Jones et al., 2021; Yuan et al., 2021). These estimates may however conflate the effects of EA with genetic nurture – a phenomenon where parental genotypes influence offspring outcomes through environmental pathways, independent of the child’s own genetic makeup (Brumpton et al., 2020; Kong et al., 2018; Morris et al., 2020). For instance, parents with a genetic predisposition to higher EA may provide resources (e.g., cognitive stimulation, financial stability), which promote their children’s educational success and mental well-being.

Additionally, population-based MR estimates are likely biased by demographic factors (e.g., assortative mating and population structure) (Brumpton et al., 2020). Bias related to assortative mating (i.e., non-random matching between reproductive partners) can arise when assortment leads to a genetic correlation between parents, which induces spurious genotype–phenotype associations in the offspring (Border et al., 2022; Hartwig et al., 2018). Bias related to population structure (i.e., the presence of systematic differences in allele frequencies between subpopulations) can occur when ancestry is correlated with both the genotype (e.g., EA genotype) and the phenotype (e.g., MHC) (Brumpton et al., 2020).

The objective of the present study is to evaluate the extent to which the associations between EA and MHC, specifically symptoms of anxiety, depression, and neuroticism, along with psychotropic medication use, are accounted for by genetic nurture and demographic factors. To address this, we applied a within-sibship MR design, which accounts for genetic nurture and reduces bias from assortative mating and population structure.

## Methodology

### Study design and data sources

The present is the report of a within-sibship MR study. MR assumptions and how they have been addressed are summarized in eTable 1. One-sample and two-sample MR methods were applied using individual-level data, and summary statistics from genome wide association studies (GWAS). Individual-level data came from the Trøndelag Health Study (HUNT) and UK Biobank (UKB) (Bycroft et al., 2018; Olav et al., 2022). A brief description of contributing GWAS can be found in eTable 2.

### Setting and participants

HUNT is a population-based cohort study that is held in the Trøndelag County in Norway and started in 1984 (Olav et al., 2022). We used data from all participants of the second (HUNT2) and third HUNT wave (HUNT3) who had been genotyped. From those, we selected all individuals who were > 30 years of age when they participated in the survey, and had at least one sibling. The final sample from HUNT2 and HUNT3 included 26 770 (10 428 sibships) and 16 718 siblings (7010 sibships), respectively (eTable 3).

The UKB is a prospective cohort study that began in 2006. UKB is following nearly 500 000, 40- to 69-year-old participants from across the UK, who volunteered to be part of the study and provided consent for follow-up through linkage to their health records (Bycroft et al., 2018). We included all participants who had been genotyped. After restricting the sample to sibships with two or more individuals, our analysis sample included 35 118 participants from 17 079 sibships (eTable 3).

### Genetic variants

For HUNT participants, we used a weighted PGS as an instrumental variable for EA (PGS-edu). The calculation of the PGS-edu was based on the genetic variants reported as significantly associated with years of education at the genome-wide level (*p* < 5 × 10^−8^) in a recent GWAS (Okbay et al., 2022). From the 3952 independent genetic variants reported by Okbay et al. (pairwise r^2^ = 0.1, no physical distance cut-off), we included those that were well imputed in the target population (eTable 4) (Choi et al., 2020). For further information, see eMethods.

### Exposure and outcomes

EA was the exposure of interest and was assessed through the question: For HUNT participants: ‘What is your highest level of education?’ For UKB participants ‘What qualifications do you have?’ Number of years of education was assigned for each of the answers based on The International Standard Classification of Education (ISCED) mapping 1997 (eTable 5). Symptoms of anxiety, depression and neuroticism, as well as the use of psychotropic medication, were the outcomes of interest. In HUNT, symptoms of anxiety and depression were assessed by the Hospital Anxiety and Depression Scale (HADS), and in UKB by the 7-item Generalized Anxiety Disorder Scale (GAD-7) and 9-item Patient Health Questionnaire (PHQ-9) (Kroenke et al., 2001; Spitzer et al., 2006; Zigmond & Snaith, 1983). In both HUNT and UKB, neuroticism was measured using the Eysenck Personality Questionnaire and use of psychotropic medication was self-reported (Eysenck & Tambs, 1990). A detailed description of how the exposure and outcomes were processed can be found in eMethods.

### Ethics approval and informed consent

The study protocol was approved by the Regional Committees for Medical Research Ethics South East (REK 2017/2479) and Mid-Norway (REK 2015/1197). All participants signed informed consent for participation and the use of data in research. UK Biobank obtained ethics approval from the North West Multi-centre Research Ethics Committee and obtained informed consent from all study participants.

### Statistical analysis

Before running the analyses using individual-level data, we standardized all numerical variables so that they had a mean of 0 and a standard deviation (SD) of 1. EA, as well as symptoms of anxiety, depression, and neuroticism, were analyzed as continuous, while use of psychotropic medication was analyzed as categorical (yes/no). We estimated the association of EA and the outcomes using one-sample MR (two-stage least squares regression), and ordinary least squares (OLS) or logistic regression for comparison.

All models were adjusted by sex and age. However, when the PGS-edu was included as a predictor, the model was also adjusted by the first 10 principal components of ancestry (PCA) to account for population structure and genotyping batch. In all cases, we assumed that standard errors were correlated within sibships and therefore clustered standard errors were computed using the “vcov = cluster” command. OLS, logistic, and one-sample MR analyses were performed using the ‘feols’ (for continuous) and ‘felgm’ (for categorical) functions of the ‘fixest’ package in R (Bergé, 2018; R Core Team, 2021).

Any difference between families due to genetic nurture, assortative mating, and population structure was accounted for by using family fixed effects (anxiety, depression, and neuroticism) or the sibling difference method (psychotropic medication use) (Brumpton et al., 2020). All analyses were performed using the “fixest” package in R (see eMethods for a detailed description) (Bergé, 2018).

Individual-level data for the two cohorts were analyzed separately using the same model specifications and R packages. Then, results were meta-analyzed using the ‘rma’ function from the ‘metafor’ R package (Viechtbauer, 2010). We applied fixed effect models, except when heterogeneity between HUNT and UK Biobank estimates was detected, that is Cochran’s Q Chi^2^ < 0.05 and I^2^ > 50% (eTable 6).

Two-sample MR analyses were performed using summary statistics from GWAS described in eTable 2 and the R package ‘TwoSampleMR’ (Hemani et al., 2018). The inverse-variance estimator weighted (IVW) estimator and its 95% confidence intervals (CI) are reported in the present work. Pleiotropy robust estimators, including weighted median, weighted mode, and MR-Egger, were used to investigate pleiotropy and reported in Supplementary Tables. The directionality of the effect was evaluated using the Steiger test of directionality. The MR-Egger intercept test was performed to assess pleiotropy. We only had access to summary statistics from within-sibship GWAS meta-analysis for depressive symptoms and neuroticism to conduct within-sibship two-sample MR (eTable 2) (Howe et al., 2021).

### Handling of missing information

We imputed missing data for various questions of the HADS score, education, and psychotropic medication use in HUNT (see eTable 7 for details on missing information). We applied multivariate imputation to each HUNT survey, using the conditional specification implemented by the MICE algorithm (van Buuren & Groothuis-Oudshoorn, 2011). For each imputed data set, we then calculated the corresponding score. For further information, see eMethods.

## Results

### Descriptive statistics

In both cohorts, participation rates were higher among females than among males. HUNT participants were younger and had slightly lower mean education years than UKB participants (13 versus 14 years). HUNT participants more frequently reported symptoms of anxiety than UKB participants (8% versus 3%). About 5% of HUNT and UKB participants reported symptoms of depression (Table 1). Psychotropic medication use was slightly higher among HUNT than UKB participants (9% versus 8%). Differences between females and males can be seen in Table 1. For each cohort, the total number of siblings varied depending on the outcome (eTable 2).

**Table 1.** General characteristics of HUNT and UKB samples

	HUNT			UK Biobank		
	Female 13 814 (51.60)	Male 12 956 (48.40)	Total 26 770	Female 20 278 (57.74)	Male 14 840 (42.26)	Total 35 118
Education (years)						
Median (IQR)	10 (10, 13)	13 (10, 13)	13 (10, 13)	10 (10, 20)	13 (10, 20)	13 (10, 20)
Mean (SD)	12.28 (2.89)	12.71 (2.93)	12.49 (2.91)	13.22 (5.00)	13.95 (5.19)	13.53 (5.09)
Age (years)						
Median (IQR)	51.50 (42, 65)	51.30 (42, 64)	51.40 (42, 65)	58 (52, 63)	58 (52, 63)	58 (52, 63)
Mean (SD)	53.61 (14.31)	53.25 (13.80)	53.43 (14.07)	56.89 (7.28)	57.28 (7.42)	57.05 (7.34)
Anxiety						
Median (IQR)	4 (2, 7)	3 (2, 6)	4 (2, 6)	1 (0, 3)^a^	0 (0, 2)^a^	0 (0, 3)^a^
Mean (SD)	4.60 (3.51)	3.85 (3.11)	4.24 (3.34)	2.14 (3.22)^a^	1.56 (2.81)^a^	1.91 (3.08)^a^
> 10, n (%)	1325 (9.59)	717 (5.53)	2042 (7.63)	108 (3.65)^a^	56 (2.94)^a^	164 (3.37)^a^
Depression						
Median (IQR)	3 (1, 5)	3 (1, 6)	3 (1, 6)	2 (0, 4)^a^	1 (0, 3)^a^	2 (0, 4)^a^
Mean (SD)	3.63 (3.07)	3.87 (3.09)	3.75 (3.08)	2.79 (3.56)^a^	2.41 (3.55)^a^	2.64 (3.56)^a^
> 10, n (%)	717 (5.19)	734 (5.67)	1451 (5.42)	165 (5.58)^a^	93 (4.88)^a^	258 (5.31)^a^
Neuroticism						
Median (IQR)	1.80 (0, 3)^b^	1 (0, 2)^b^	1 (0, 3)^b^	4 (2, 7)^c^	3 (1, 6)^c^	4 (1, 6)^c^
Mean (SD)	1.94 (1.82)^b^	1.31 (1.55)^b^	1.65 (1.73)^b^	4.49 (3.22)^c^	3.49 (3.18)^c^	4.07 (3.24)^c^
Psych. med.						
n (%)	1604 (11.61)	784 (6.05)	2388 (8.92)	2038 (10.05)	896 (6.04)	2934 (8.35)

*Note:* In HUNT symptoms of anxiety and depression were assessed by the Hospital Anxiety and Depression Scale (HADS), and in UK Biobank by the 7-item Generalized Anxiety Disorder Scale (GAD-7) and 9-item Patient Health Questionnaire (PHQ-9), respectively. Neuroticism was assessed using a six-item and a 12-item Eysenck Personality Questionnaire in HUNT and UK Biobank, respectively (see eMethods).

Abbreviations and symbology: n, number; %, percentage; IQR, interquartile range; SD, standard deviation; Psych. med., psychotropic medication usage; a, assessed in 4863 participants (n female = 2959 (60.85%), n male = 1904 (39.15%)); b, assessed in 16 718 participants (n female = 8965 (53.62%), n male = 7753 (46.38%)); c, assessed in 23 852 participants (n female = 13 664 (57.29%), n male = 10 188 (42.71%)).

The PGS-edu was associated with years of education, conditional from age, sex, first 10 PCA, and batch in both cohorts. In HUNT, one SD increase in the PGS-edu was associated with a 0.19 SD (~0.55 years) increase in years of education (_95%_CI, 0.18: 0.20, *p =* 4.40×10^−187^, *F-test stat.* = 162.99, *r^2^* = 0.03). In UK Biobank, each SD increase of the PGS-edu was associated with a 0.24 SD (~1.22 years) increase in years of education (_95%_CI, 0.23: 0.24, *p* = 2.20×10^−16^, *F-test stat.* = 103.90, *r^2^ =* 0.06). This association was attenuated after including a family fixed effect (HUNT: 0.13, _95%_CI: 0.11–0.15, *p =* 3.47×10^−44^, *F-test stat.* = 1349.62, *r^2^* = 0.64; UK Biobank: 0.13, _95%_CI, 0.11: 0.14, *p* = 3.38×10^−43^, *F-test stat.* = 2362.40, *r^2^* = 0.33). The associations between the PGS-edu and the outcomes are depicted in eTable 8.

### Main analyses

The direction of the regression and MR estimates was consistent across both cohorts and all analyses. However, there were some differences in association strength between HUNT and UKB (Table 2). Differences were more pronounced for the depression and neuroticism MR estimates. There was little evidence of weak instrument bias, as all F-stats were higher than 10 (eTable 9).

**Table 2.** Association between educational attainment and mental health outcomes

Outcome	HUNT					UK Biobank					Meta-analysis				
	B	LCI	UCI	SE	p	B	LCI	UCI	SE	p	B	LCI	UCI	SE	p
Anxiety															
OLS EA	−0.07	−0.08	−0.06	0.01	2.68×10^−25^	−0.02	−0.05	0.00	−0.02	0.090	−0.06	−0.11	−0.01	0.03	0.017
OLS EA + FE	−0.05	−0.07	−0.03	0.01	6.03×10–^07^	−0.02	−0.07	0.02	−0.02	0.286	−0.04	−0.06	−0.03	0.01	5.49×10^−07^
1SMR	−0.20	−0.26	−0.13	0.04	3.07×10^−08^	−0.22	−0.34	−0.10	−0.22	2.99×10^−04^	−0.20	−0.26	−0.14	0.03	1.89×10^−10^
1SMR + FE	−0.08	−0.24	0.08	0.08	0.347	−0.65	−1.25	−0.04	−0.65	0.036	−0.17	−0.33	−0.00	0.08	0.049
Depression															
OLS EA	−0.09	−0.10	−0.07	0.01	5.52×10^−40^	−0.06	−0.09	−0.03	−0.06	2.05×10^−05^	−0.08	−0.09	−0.07	0.01	2.34×10^−44^
OLS EA + FE	−0.06	−0.08	−0.04	0.01	1.76×10^−10^	−0.02	−0.07	0.02	−0.02	0.328	−0.06	−0.07	−0.04	0.01	4.13×10^−10^
1SMR	−0.06	−0.13	0.00	0.03	0.058	−0.34	−0.47	−0.21	−0.34	1.20×10^−07^	−0.11	−0.43	0.22	0.17	0.518
1SMR + FE	−0.03	−0.20	0.13	0.08	0.674	−1.00	−1.68	−0.31	−1.00	0.004	−0.18	−1.26	0.89	0.55	0.739
Neuroticism															
OLS EA	−0.11	−0.13	−0.09	0.01	6.94×10^−33^	−0.08	−0.09	−0.06	0.01	6.93×10^−31^	−0.09	−0.12	−0.06	0.02	1.21×10^−07^
OLS EA + FE	−0.07	−0.09	−0.04	0.01	6.21×10^−08^	−0.04	−0.06	−0.02	0.01	1.01×10^−04^	−0.05	−0.08	−0.02	0.01	5.96×10^−04^
1SMR	−0.44	−0.53	−0.34	0.05	3.89×10^−18^	−0.20	−0.25	−0.14	0.03	7.96×10^−13^	−0.30	−0.53	−0.06	0.12	0.015
1SMR + FE	−0.40	−0.62	−0.17	0.11	4.44×10^−04^	−0.22	−0.40	−0.04	0.09	0.016	−0.29	−0.43	−0.15	0.07	3.64×10^−05^
Psych. med.*															
LOG EA	0.85	0.79	0.90	0.03	1.72×10^−07^	0.63	0.53	0.74	0.08	4.37×10^−08^	0.85	0.82	0.88	0.02	2.85×10^−19^
LOG EA + FE	0.90	0.82	0.99	0.05	0.027	0.47	0.25	0.87	0.31	0.017	0.93	0.88	0.98	0.03	0.007
1SMR	0.56	0.44	0.71	0.12	1.93×10^−06^	0.85	0.82	0.89	0.02	3.41×10^−14^	0.60	0.52	0.69	0.07	5.31×10^−13^
1SMR + FE	0.60	0.32	1.14	0.33	0.120	0.95	0.89	1.01	0.03	0.116	0.52	0.34	0.82	0.23	0.004

*Note:* HUNT and UK Biobank data were analyzed separately and results then meta-analyzed (see Methodology).

Abbreviations and symbology: B, coefficient; SE, standard error; LCI, low 95% confidence interval; UCI, upper 95% confidence interval; *p*, p value; n, number; OLS, ordinary least squares regression; LOG, logistic regression; EA, educational attainment; FE, within-sibship adjustment; PGS-edu, educational attainment polygenic score; 1SMR, one-sample Mendelian randomization; Psych. med., psychotropic medication usage; *, coefficient and confidence intervals were exponentiated and hence odd ratios are presented.

**Figure 1.**
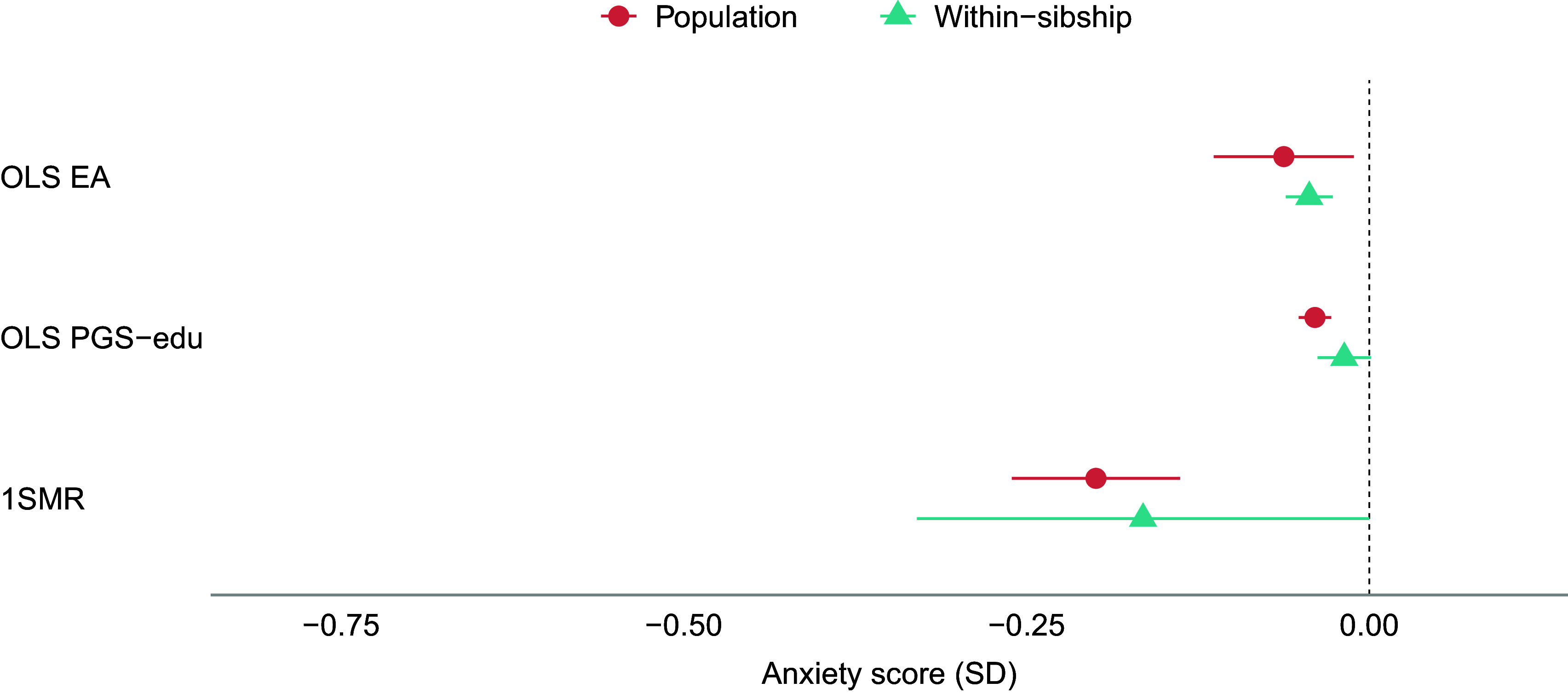
Educational attainment and symptoms of anxiety. Standard deviation (SD) changes in the anxiety score and its 95% confidence interval per SD increase in years of education are shown. Estimated associations are displayed for ordinary least squares regression (OLS) and Mendelian randomization models. Note: SD, ‘standard deviation unit’; OLS EA, ‘ordinary least squares regression model with educational attainment as exposure’; OLS PGS-edu, ‘ordinary least squares regression model with the educational attainment polygenic score as exposure’; 1SMR, ‘one-sample Mendelian randomization’.

**Figure 2.**
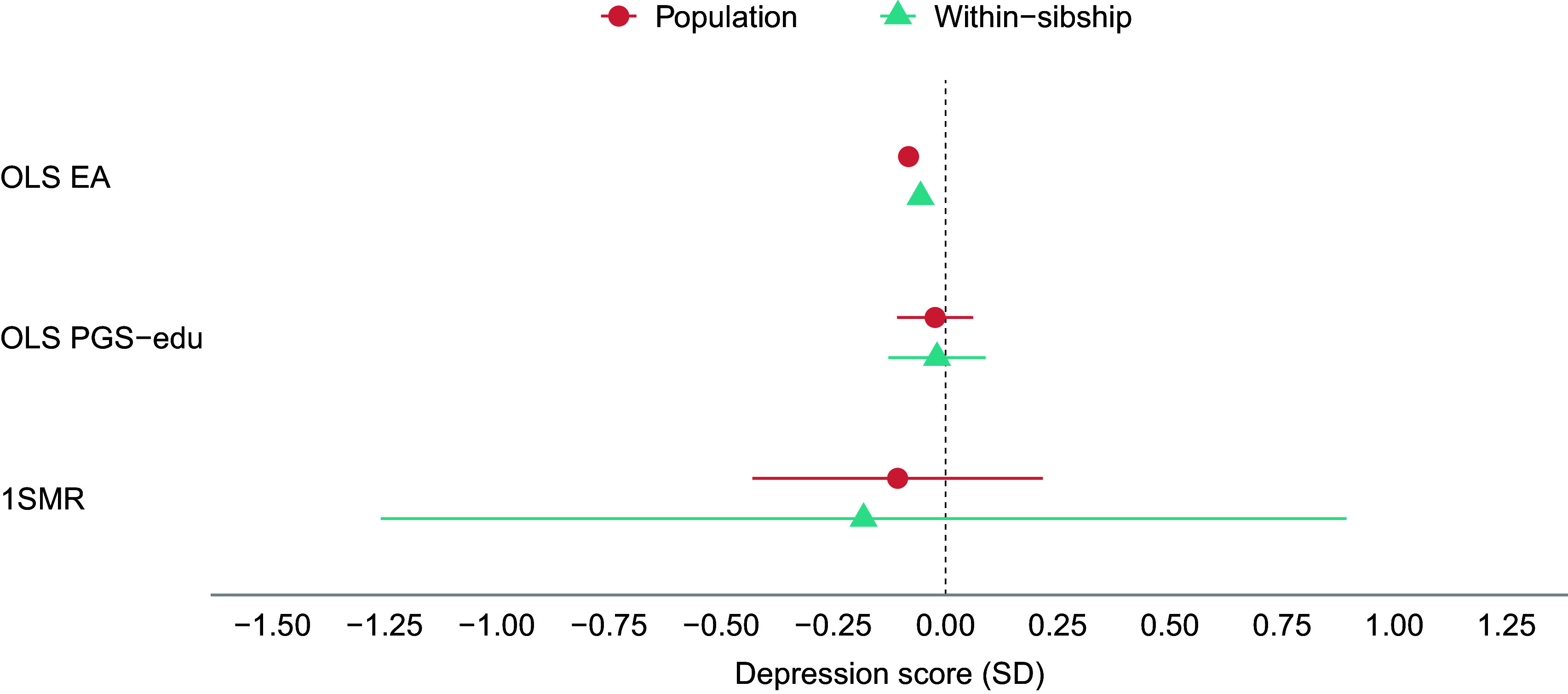
Educational attainment and symptoms of depression. Standard deviation (SD) changes in the depression score and its 95% confidence interval per SD increase in years of education are shown. Estimated associations are displayed for ordinary least squares regression (OLS) and Mendelian randomization models. Note: SD, ‘standard deviation unit’; OLS EA, ‘ordinary least squares regression model with educational attainment as exposure’; OLS PGS-edu, ‘ordinary least squares regression model with the educational attainment polygenic score as exposure’; 1SMR, ‘one-sample Mendelian randomization’; 2SMR, ‘two-sample Mendelian randomization’.

**Figure 3.**
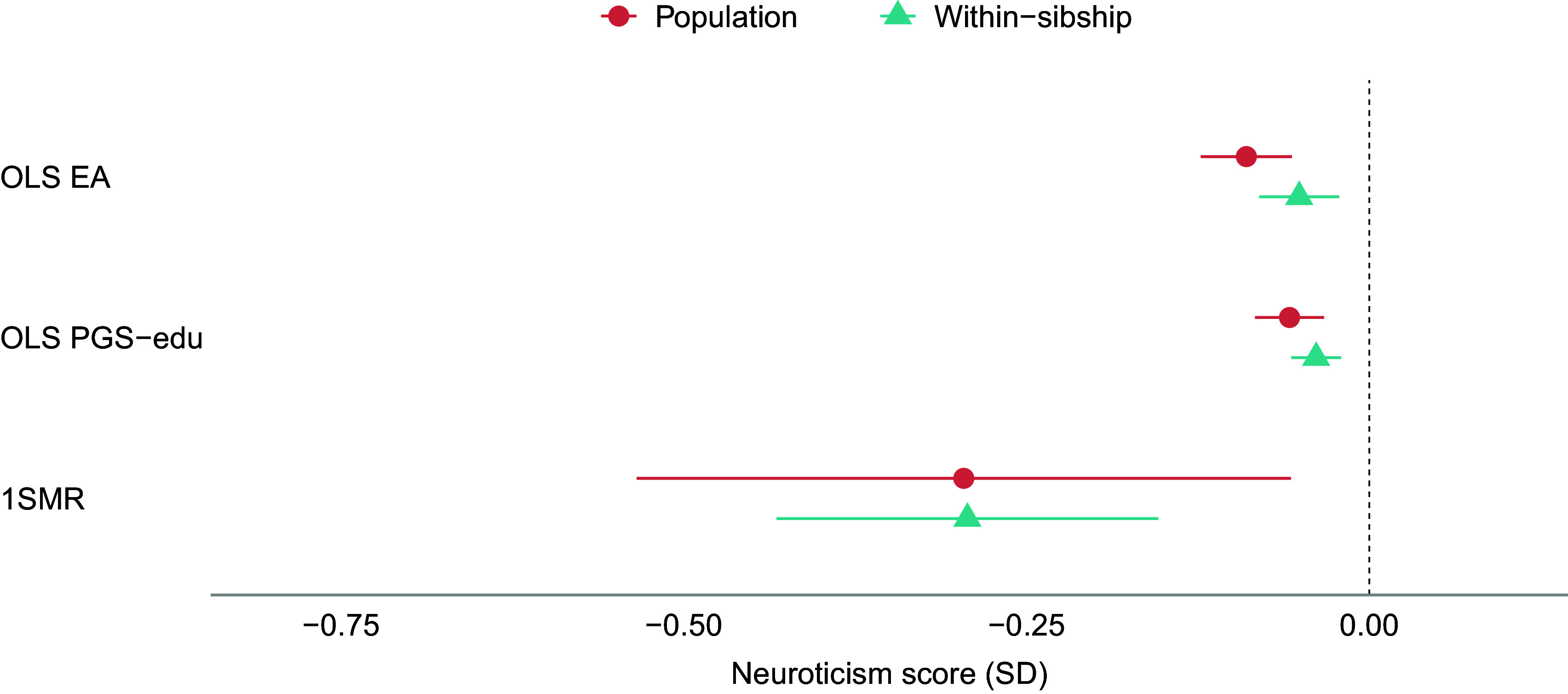
Educational attainment and neuroticism. Standard deviation (SD) changes in the neuroticism score and its 95% confidence interval per SD increase in years of education are shown. Estimated associations are displayed for ordinary least squares regression (OLS) and Mendelian randomization models. Note: SD, ‘standard deviation unit’; OLS EA, ‘ordinary least squares regression model with educational attainment as exposure’; OLS PGS-edu, ‘ordinary least squares regression model with the educational attainment polygenic score as exposure’; 1SMR, ‘one-sample Mendelian randomization’; 2SMR, ‘two-sample Mendelian randomization’.

**Figure 4.**
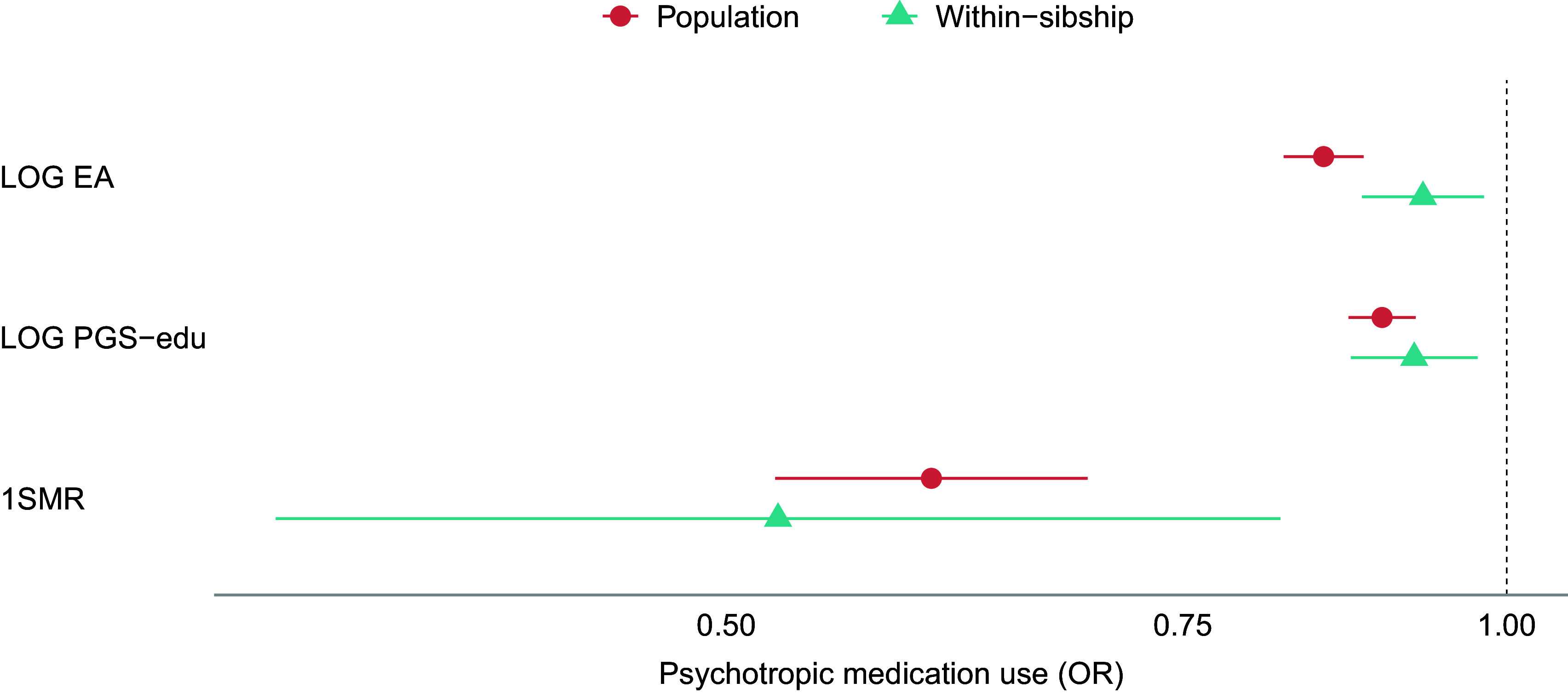
Educational attainment and use of psychotropic medication. Log odds changes in psychotropic medication use and its 95% confidence interval per SD increase in years of education are shown. Estimated associations are displayed for logistic regression (LOG) and Mendelian randomization models. Note: SD, ‘standard deviation unit’; LOG EA, ‘logistic regression model with educational attainment as exposure’; LOG PGS-edu, ‘logistic regression model with the educational attainment polygenic score as exposure’; 1SMR, ‘one-sample Mendelian randomization’.

The results of the population-based MR analyses indicate that a genetic liability to higher EA has protective effects on anxiety, neuroticism, and psychotropic medication use (Figures 1, 3, and 4). A one SD increase in genetic liability to EA was associated with reduced anxiety (−0.20 SD [−0.26, −0.14]), neuroticism scores (−0.30 SD [−0.53, −0.06]), and lowered the odds of psychotropic medication use (odds ratio (OR): 0.60 [0.52, 0.69]). For depression, MR estimates included the null (−0.11 SD [−0.43, 0.22]) (Figure 2). The one-sample MR estimates were larger than the phenotypic regression estimates, except for depression, but were consistent in direction (Table 2).

The within-sibship MR estimates were generally consistent with the population-based MR estimates (Table 2 and Figures 1–4). Although some changes were observed, within-sibship estimates overlapped with population-based MR estimates (Figures 1–4). Specifically, the effect of the genetic liability to higher EA on anxiety changed from −20 SD to −0.17 SD [−0.33, −0.00], on depression from −11 SD to −18 SD [−1.26, 0.89], and on neuroticism from −0.30 SD to −0.29 SD [−0.43, −0.15]. The OR for psychotropic medication use decreased from 0.60 to 0.52 [0.34, 0.82].

Two-sample MR estimates for depression and neuroticism were consistent in direction with those from one-sample MR (eTable9). A one SD increase in genetic liability to EA reduced depression and neuroticism scores by (−0.22 [−0.25, −0.18]) and (−0.21 [−0.25, −0.17]), respectively. Also, the weighted median, weighted mode, and MR-Egger estimates were consistent with IVW estimates. In both cases, the directionality test indicated that the causal direction is likely correct and that horizontal pleiotropy is unlikely (eTable 10). Within-sibship two-sample MR estimates were consistent with population-based estimates but were less precise, as the depression estimate including the null hypothesis.

## Discussion

Our phenotypic and population-based one-sample MR analyses indicated that a genetic liability to higher EA reduces symptoms of anxiety and neuroticism and lowers psychotropic medication use. Complementary population-based two-sample MR analyses further indicated that a genetic liability to higher EA reduces depression symptoms. When accounting for genetic nurture and demographic factors, effect estimates changed slightly and became less precise but remained consistent with the population-based estimates. This implies that these associations are unlikely to be attributable to genetic nurture linked to EA or demographic factors. Overall, these findings suggest that higher EA (its genetic liability or some other closely related traits) reduces the risk of developing these MHC in adulthood.

In line with our results, previous research found little evidence for genetic nurture linked to parental EA influencing children’s depression, anxiety, and attention-deficit hyperactivity symptoms (Hughes et al., 2024). Hughes et al. reported that children’s PGS-Edu were negatively associated with these traits, independent of genetic nurture, implying that liability to higher EA may protect against MHC. In contrast, another study using the same cohort identified broad parental genetic nurture effects on depression (i.e., not specifically related to EA), which were partially mediated via parental anxiety and depression symptoms (Cheesman et al., 2020). These results suggest that while genetic nurture linked to EA likely does not contribute to MHC risk, genetic nurture tied to other parental traits (e.g., mental health) could still play a role.

While the magnitude of change in effect estimates varied across the examined MHC, it was generally modest. Moreover, all within-sibship MR effect estimates displayed wide confidence intervals that overlapped with the population-based MR estimates. The degree of imprecision was most pronounced for depression, for which within-sibship MR estimates also overlapped with the null hypothesis. Given this uncertainty, we cannot exclude a potential effect of EA on adult depression symptoms. In contrast, a recent study using a similar approach reported complete attenuation of the effects of EA on depression, anxiety, and neuroticism (van de Weijer et al., 2024). However, like our findings with respect to depression, their imprecise confidence intervals preclude definitive conclusions about the influence of EA on depression.

Importantly, in MR studies on EA, genetic instruments serve as proxies for liability to EA, rather than direct measures of educational duration. Consequently, MR estimates are unlikely to reflect the pure effect of an additional year of schooling (Howe et al., 2022). That is, the genetic liability to higher EA likely operates through both measured EA (e.g., postgraduate qualifications) and other closely related traits. For instance, the PGS-edu likely influences mental health not only by increasing the likelihood of education achievement, but also by shaping unmeasured characteristics like personality or cognitive ability (Krapohl et al., 2014). Furthermore, the phenotypic expression of the genetic liability to higher EA depends on social, historical, and cultural contexts, implying that *its impact on the risk of developing MHC may differ across populations* (Border et al., 2022; Okbay et al., 2022; Rutherford et al., 2025).

### Strengths and limitations

A major strength of our work is that we applied robust methods to evaluate the impact of genetic nurture and demographic factors on the association between EA and the studied MHC (Brumpton et al., 2020; Howe et al., 2021). As genetic variants are randomly assigned at conception, MR studies are potentially less susceptible to bias from confounding or reverse causation. Within-family MR addresses key limitations of population-based MR, such as violations of the independence assumption (Brumpton et al., 2020). Importantly, our results were replicated in two cohorts and across different analyses: phenotypical and MR estimates were consistent in both cohorts. Nevertheless, our approach has limitations that must be considered when interpreting our findings.

Limited precision hinders definitive conclusions about whether the effect of EA on depression is explained by genetic nurture and demographic factors. This limitation likely stems from four methodological constraints. First, cohort heterogeneity between the HUNT and UKB, including instrument differences (e.g., HADS versus PHQ-9) and population characteristics (e.g., age, socioeconomic contexts), increases heterogeneity in effect estimates. Second, self-reported depression symptoms increase the likelihood of measurement error, which reduces statistical power and attenuates effect sizes. Third, while MR estimates within cohorts are robust to individual-level confounding, between-cohort heterogeneity in pooled estimates may reflect either true population differences or methodological constraints. With only two cohorts, we cannot distinguish these scenarios. Fourth, given the episodic nature of depression, which is strongly influenced by short-lived environmental effects, single time-point assessments may attenuate the observed associations between EA and depression (Kendler & Gardner, 2017).

While previous evidence supports the use of self-reported medication as an alternative or supplementary phenotype for anxiety and depression in genetic studies, an important implementation limitation warrants consideration (Skelton et al., 2021). Factors such as socioeconomic barriers to healthcare access, stigma, and regional variations in prescribing practices may introduce measurement error. This can manifest as both under-ascertainment of true medication need among individuals with MHC and misclassification of case status, thereby potentially attenuating effect estimates toward the null. Despite this limitation, we observed consistent protective effects associated with a genetic liability to higher EA across both psychotropic medication use and symptom severity measures. This convergence across multiple proxies strengthens the hypothesis that genetic liability to higher EA reduces the risk of developing these MHC in adulthood.

While within-sibship MR designs are robust to genetic nurture and demographic factors, biases may persist due to unmeasured family-level environmental influences (Brumpton et al., 2020; Howe et al., 2022). Indirect genetic effects between siblings, such as the influence of sibling genotypes on the shared environment, could explain our results (Howe et al., 2022). For example, an older sibling’s academic success raises parental expectations for younger siblings, independent of the younger siblings’ genetics. Unmeasured family-level environmental factors, such as differential parental treatment (e.g., unequal resource allocation between siblings in response to inherited variants), could further confound results (Sjölander et al., 2022). While sibling influences (including birth order dynamics) could potentially distort within-sibship MR estimates, their magnitude is expected to be smaller than major confounding pathways addressed by our design (Demange et al., 2022).

Other limitations include possible weak instrument bias. In the general population, the PGS-edu’s explanatory power (R^2^) was relatively small (~3%), which is expected to attenuate further in within-sibship analyses due to reduced within-family variation (Sjölander et al., 2022). Because instrument strength depends on both R^2^ and sample size, the instrument’s effective strength is often diminished in within-sibship analyses. Although our F-statistics exceeded conventional thresholds for instrument strength (*F* > 10, see eTable 9), the low *R^2^* reflects a weak genetic signal, increasing susceptibility to weak instrument bias. Assuming that our PGS-edu is a valid instrument, effect estimates would be biased toward the null, potentially masking a true protective effect of EA. This limitation is particularly critical for interpreting the effect of EA on depression symptoms compared to other outcomes, as it could obscure a true protective effect of a genetic liability to higher EA on depression.

Selection bias is a recognized limitation of the UK Biobank, as participants are non-randomly sampled and over-represent healthier, wealthier, and more educated individuals (Tyrrell et al., 2021). While simulations suggest selection bias may have less impact on MR than pleiotropy or population stratification, it may still differentially influence our analyses (Gkatzionis & Burgess, 2019). In within-sibship, MR, non-random participation within families, such as the systematic enrolment of siblings with higher education or better health, may induce selection or collider bias (Sjölander et al., 2022). Additionally, as MR analyses rely on available GWAS data, our study is constrained by the lack of ancestral and geographic diversity, which limits generalizability. These limitations highlight the need for replication in more representative cohorts.

## Conclusions

Our study suggests that higher EA (or genetic liability to education) may help reduce anxiety, neuroticism, and psychotropic medication use. These mental health benefits do not seem to be explained by EA-linked genetic nurture or demographic factors (e.g., assortative mating, population structure). Regarding depression, results were inconclusive due to imprecise estimates, though beneficial effects of genetic liability to higher EA are possible and warrant further investigation. Additionally, future research should investigate how education (or other closely related phenotypes, such as cognitive skills and income) impacts mental health across diverse populations. Broader areas of research might include: (1) Examining mediating pathways (e.g., mental health literacy and improved living conditions); (2) Identifying critical developmental windows; (3) Understanding gene–environment interplay; and (4) Developing and testing interventions for high-risk groups.

## Supporting information

Vinueza Veloz et al. supplementary material Vinueza Veloz et al. supplementary material

## Data Availability

Researchers associated with Norwegian research institutes can apply for the use of HUNT material: data and samples – given approval by a Regional Committee for Medical and Health Research Ethics. Researchers from other countries can also apply in cooperation with a Norwegian principal investigator. Information regarding data access can be found at https://www.ntnu.edu/hunt/data. UK Biobank individual-level participant data are available via enquiry to access@ukbiobank.ac.uk. All GWAS summary statistics used in the present manuscript are publicly available and can be downloaded from https://gwas.mrcieu.ac.uk/ and https://thessgac.com/papers/.

## Supplementary material

The supplementary material for this article can be found at http://doi.org/10.1017/S0033291725101736.

## Information on the author’s access to data

MFVV and LB had full access to all of the study’s data and are responsible for its integrity and the accuracy of the data analysis.
